# Biomechanical study on fixation methods for horizontal oblique metacarpal shaft fractures

**DOI:** 10.1186/s13018-022-03267-2

**Published:** 2022-08-03

**Authors:** Yung-Cheng Chiu, Cheng-En Hsu, Tsung-Yu Ho, Yen-Nien Ting, Ming-Tzu Tsai, Jui-Ting Hsu

**Affiliations:** 1 School of Medicine, China Medical University, Taichung, 404 Taiwan; 2 Department of Orthopedic Surgery, China Medical University Hospital, Taichung, 404 Taiwan; 3 Department of Orthopaedics, Taichung Veterans General Hospital, Taichung, 407 Taiwan; 4 Sports Recreation and Health Management Continuing Studies-Bachelor’s Degree Completion Program, Tunghai University, Taichung, 407 Taiwan; 5 3D Printing Medical Research Center, China Medical University Hospital, Taichung, 404 Taiwan; 6 Department of Biomedical Engineering, Hungkuang University, Taichung, 433 Taiwan; 7 Department of Biomedical Engineering, College of Biomedical Engineering, China Medical University, Taichung, 404 Taiwan; 8 School of Dentistry, College of Dentistry, China Medical University, Taichung, 404 Taiwan; 9 Department of Bioinformatics and Medical Engineering, Asia University, Taichung, 413 Taiwan

**Keywords:** Horizontal oblique metacarpal shaft fracture, Screw, Lag screw, Regular plate, Locking plate

## Abstract

**Objective:**

To investigate differences in the effectiveness of two lag screws, a regular bone plate, and locking bone plate fixation in treating horizontal oblique metacarpal shaft fractures.

**Materials and methods:**

Horizontal oblique metacarpal shaft fractures were created in 21 artificial metacarpal bones and fixed using one of the three methods: (1) two lag screws, (2) a regular plate, and (3) a locking plate. All the specimens were subjected to the cantilever bending test performed using a material testing machine to enable recording of the force–displacement data of the specimens before failure. The Kruskal–Wallis test was used to compare failure force and stiffness values among the three fixation methods.

**Results:**

The mean failure force of the two lag screw group (78.5 ± 6.6 N, mean + SD) was higher than those of the regular plate group (69.3 ± 17.6 N) and locking plate group (68.2 ± 14.2 N). However, the mean failure force did not significantly differ among the three groups. The mean stiffness value of the two lag screw group (17.8 ± 2.6 N/mm) was lower than those of the regular plate group (20.2 ± 10.5 N/mm) and locking plate group (21.8 ± 3.8 N/mm). However, the mean stiffness value did not significantly differ among the three groups.

**Conclusion:**

The fixation strength of two lag screw fixation did not significantly differ from that of regular and locking bone plate fixation, as indicated by the measurement of the ability to sustain force and stiffness.

## Introduction

Metacarpal fractures are not uncommon and account for approximately 40% of all hand fractures [1]. Most metacarpal bone fractures can be treated conservatively [2, 3]. However, for complex fractures with comminution or unstable metacarpal fractures, such as oblique fractures, spiral fractures, or those involving the shortening of the metacarpal bone due to overlapping fracture ends, surgical intervention is required to prevent subsequent complications [4, 5]. Few studies have specifically focused on the clinical treatment of oblique metacarpal bone fractures. Currently, the following surgical methods are the most commonly used in clinical practice to treat oblique metacarpal shaft fractures: (1) lag screw fixation, (2) bone plate fixation, and (3) K-wire fixation [6]. In K-wire fixation, the fixation strength is insufficient to withstand the torsion load at the fracture site; this can lead to the rotational malunion of the fracture and eventually to a scissoring deformity. Therefore, most hand surgeons do not consider K-wire fixation as the primary treatment for oblique metacarpal fractures [7, 8]. No consensus has yet been reached regarding whether lag screw or bone plate fixation is the most favorable surgical method [6, 9]. Lag screw fixation is a minimally invasive surgical procedure. However, surgeons are often concerned lag screw fixation will be inadequately strong [10–13]. Başar et al. [14] recommended lag screw fixation only for oblique phalangeal bone fractures and bone plate fixation for oblique metacarpal bone fractures. In their biomechanical study, Adams et al. [15] indicated that the use of lag screws can result in excellent fixation strength in long oblique metacarpal shaft fractures (defined as the fracture length being longer than the diameter of the metacarpal bone) [15].

To our knowledge, previous studies have not provided clear definitions for fracture classifications or suggestions for appropriate surgical treatments for oblique metacarpal shaft fractures. In this study, we proposed a new classification system for oblique metacarpal shaft fractures, conducted biochemical studies based on our classification, and identified the most favorable surgical fixation method for oblique metacarpal shaft fractures. In addition, although many studies have investigated the fixation capacity of metacarpal transverse fractures [16, 17], few have focused on oblique fractures [6]. Moreover, no study has focused on fixation methods for horizontal oblique metacarpal shaft fractures. Therefore, this study compared the effectiveness of two lag screws, regular bone plates, and locking bone plates for fixing horizontal oblique metacarpal shaft fractures.

## Materials and methods

### Definition of oblique metacarpal shaft fractures and preparation of the artificial bone specimen

**Fig. 1:**
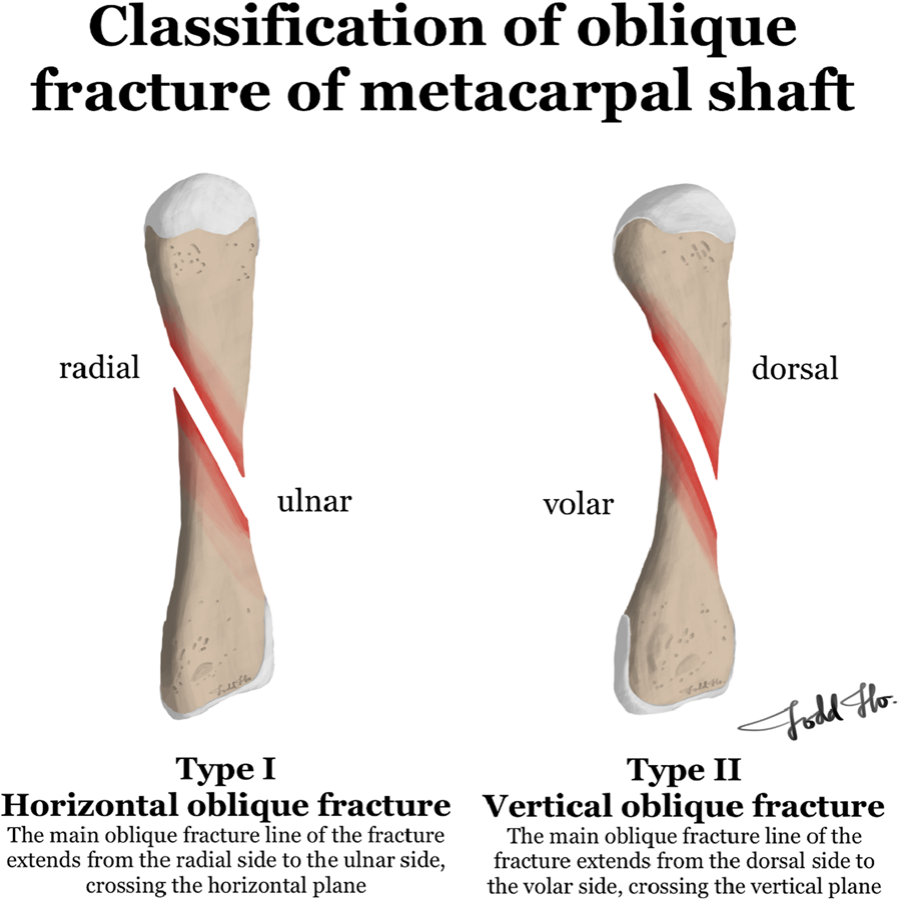
Defined oblique metacarpal shaft fracture types

We divided oblique metacarpal shaft fractures into two categories: (1) horizontal oblique fractures (type I; the main oblique fracture line extends from the radial side to the ulnar side of the metacarpal shaft, crossing the horizontal plane of the metacarpal bone) and (2) vertical oblique fractures (type II; the main oblique fracture line extends from the dorsal side to the volar side of the metacarpal shaft, crossing the vertical plane of the metacarpal bone; Fig. 1). In this study, we examined only horizontal oblique metacarpal shaft fractures (type I) and performed a biomechanical study to determine the fixation strength at fracture sites.

**Fig. 2:**
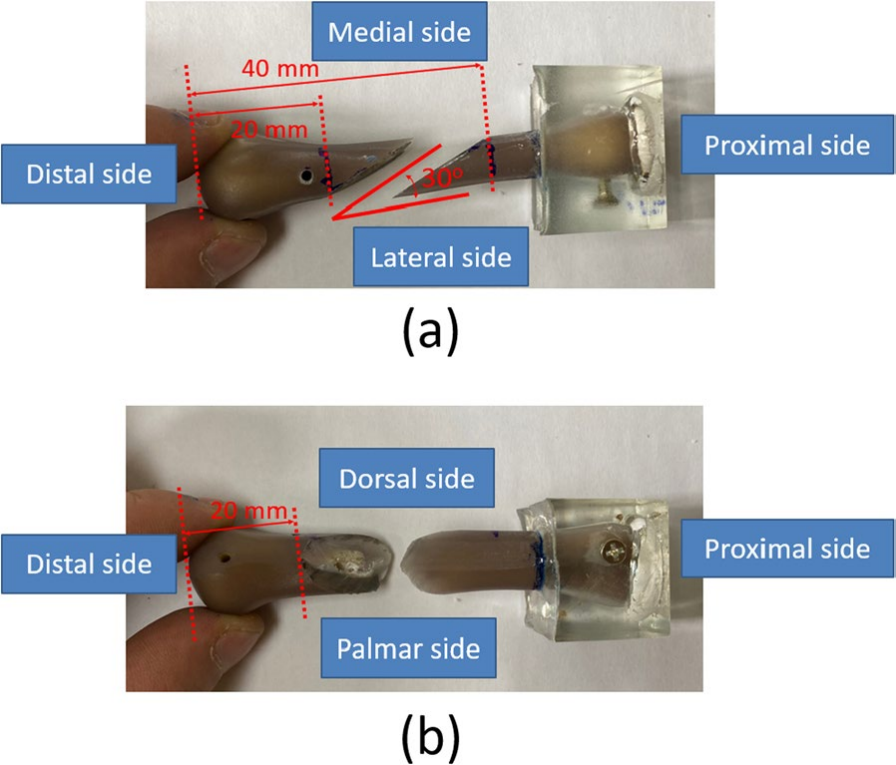
Artificial metacarpal bones with horizontal oblique metacarpal shaft fractures: **a **dorsal and **b** lateral views

A total of 21 artificial third metacarpal bones (Sawbones, Vashon, WA, USA) were used in this study. Two transverse lines were drawn first for reference. The distal line was transversely drawn across the distal metacarpal shaft approximately 20 mm from the top of the metacarpal head. The proximal line was transversely drawn across the proximal metacarpal shaft approximately 40 mm from the top of the metacarpal head. Subsequently, we connected the two parallel lines with an oblique line and used an electric saw to create a horizontal oblique fracture, with an oblique angle of 30°. Horizontal oblique metacarpal shaft fractures were created in the artificial metacarpal bones by using an electric chainsaw (Fig. 2). In addition, the proximal end of the artificial metacarpal bone was embedded in epoxy resin.

### Fixation approaches

**Fig. 3:**
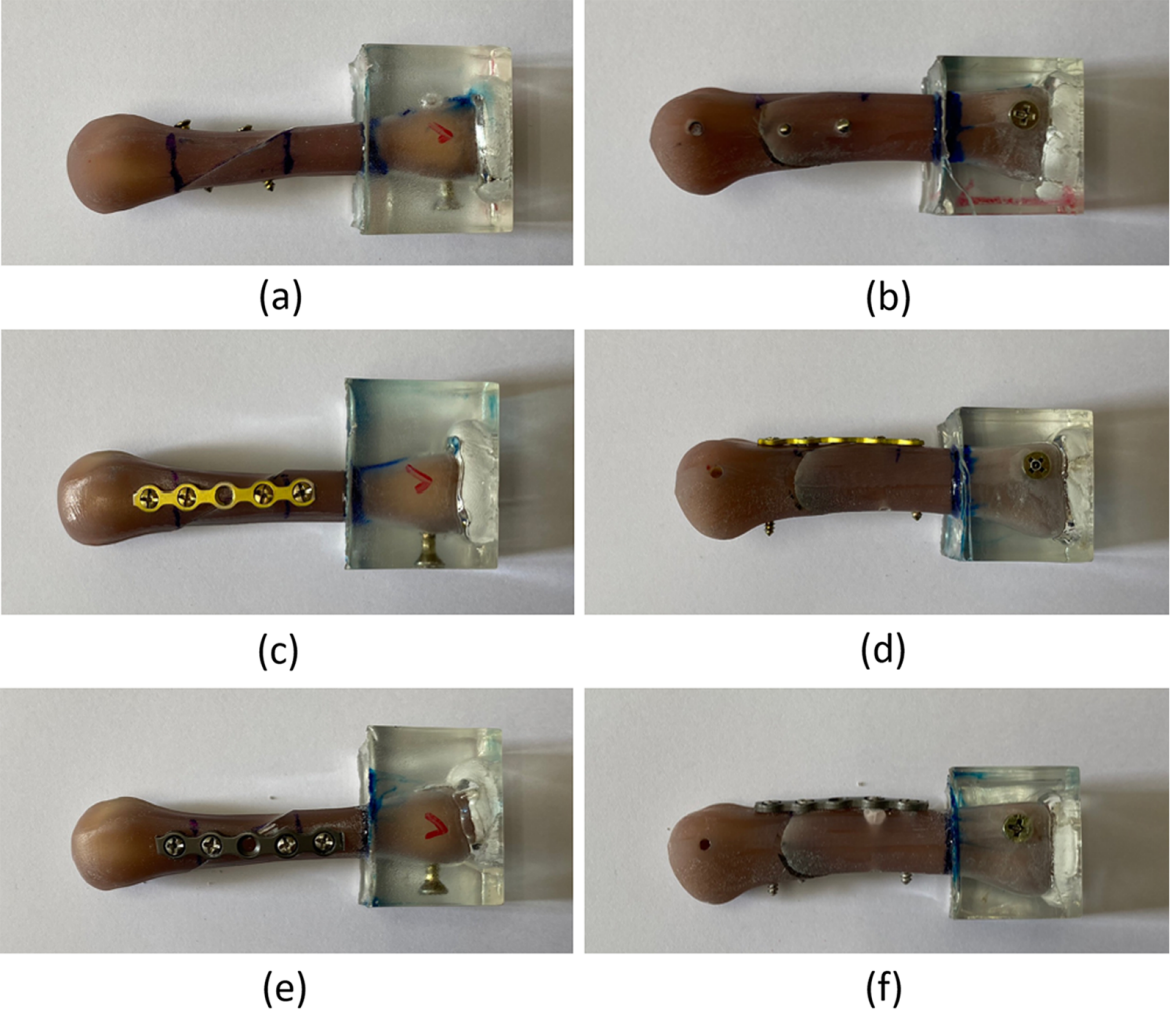
Photographs of the three fixation approaches: **a** anterior–posterior view of lag screw fixation; **b** lateral view of lag screw fixation; **c** anterior–posterior view of regular plate fixation; **d** lateral view of regular plate fixation; **e** anterior–posterior view of locking plate fixation; and **f** lateral view of locking plate fixation

Horizontal oblique metacarpal shaft fractures were created in the 21 artificial metacarpal bones by using a chainsaw. The bones were equally distributed into three groups: (1) two lag screws (LS), (2) regular plate (RP), and (3) locking plate (LP) (Fig. 3). All fracture fixation surgeries were performed by a single senior hand surgeon, Dr. Y.C. Chiu.

LS group: After the manual reduction of the artificial bone with a horizontal oblique fracture, two parallel 2.3-mm cortical screws (Stryker, Germany) were inserted from the lateral side of the cortex. The lag screws were inserted into the bones in the direction perpendicular to the fracture line. To fix the fracture, both the lag screws were inserted completely until they penetrated the contralateral cortex (distal cortex) (Fig. 3a, b).

RP group: After the manual reduction of the artificial bone with a horizontal oblique fracture, a regular 5-hole plate (steel plate model: Stryker, Germany) was applied on the dorsal cortex of the metacarpal bone. The plate was screwed to the proximal and distal ends of the fracture site by using two conventional cortical screws on each side. All four screws penetrated the distal and proximal cortical bones (Fig. 3c, d).

LP group: After the manual reduction of the artificial bone with a horizontal oblique fracture, a locking 5-hole plate (steel plate model: Stryker, Germany) was applied on the dorsal cortex of the metacarpal bone. The plate was screwed to the proximal and distal ends of the fracture site by using two conventional locking cortical screws on each side. All four screws penetrated the distal and proximal cortical bones (Fig. 3e, f).

### Biomechanical test

**Fig. 4:**
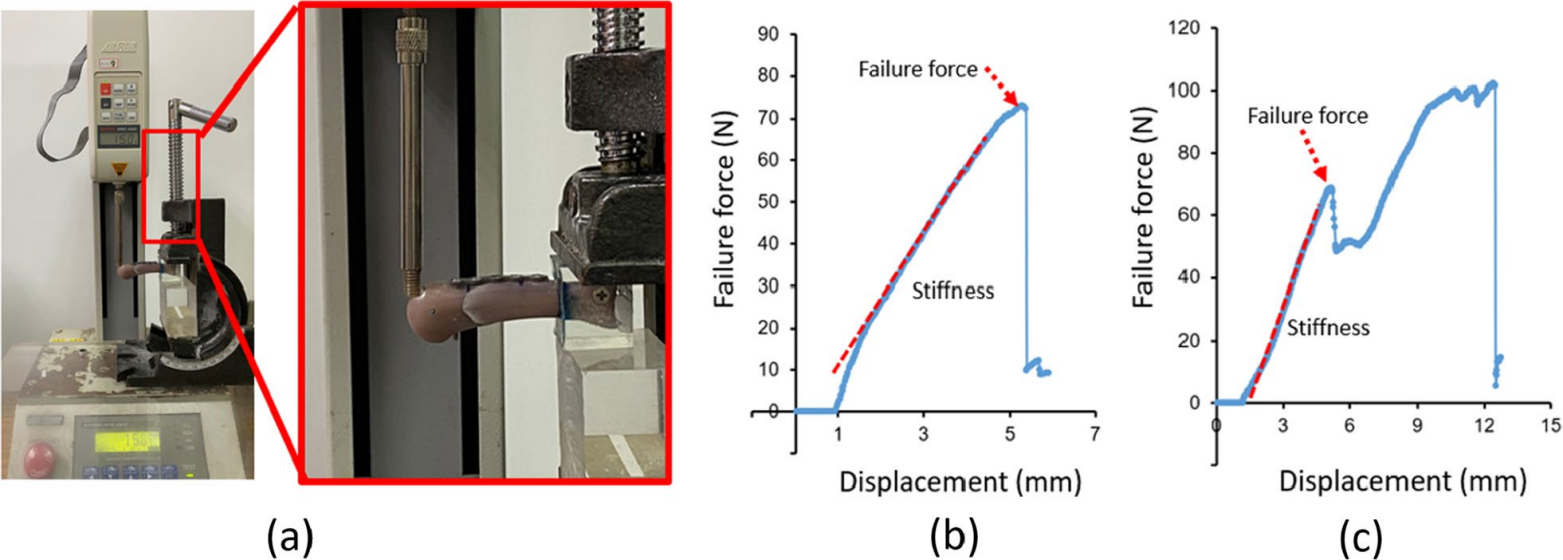
**a** Biomechanical cantilever bending test; **b** force–displacement curve of lag screw fixation; and **c** force–displacement curve of locking plate fixation

We performed the cantilever bending test in vitro. The material testing system used in this study was the JSV-H1000 (Japan Instrumentation System, Nara, Japan; Fig. 4a). At the distal region of the dorsal side of the artificial metacarpal bone, force was applied at a rate of 10 mm/min. The force–displacement curve was plotted as the force was applied, and failure force and stiffness values were determined from the plotted force–displacement curve (Fig. 4b, c).

### Statistical analysis

The failure force and stiffness values of the three fixation methods are expressed as means and standard deviations. The Shapiro–Wilk test revealed that the failure force and stiffness were not normally distributed among the three groups. Therefore, the Kruskal–Wallis test was performed to compare the effectiveness of the three methods in fixing horizontal oblique metacarpal shaft fractures. If a statistical difference was identified, post hoc pairwise comparisons were conducted by the exact test of Wilcoxon rank sum test with the Bonferroni adjustment, and the significance level was 0.0167 (0.05/3). All statistical analyses were performed using SPSS 19.0 (IBM Corporation, Armonk, NY, USA), and a *P* value of < 0.05 was considered statistically significant.

## Results

Table 1 lists the failure force and stiffness values of the three fixation methods. The mean failure force value of the LS group (78.5 ± 6.6 N) was higher than those of the RP (69.3 ± 17.6 N) (P = 0.394) and LP (68.2 ± 14.2 N) (*P* = 0.310) groups. The mean failure force of the LS group was 13.3% and 15.1% higher than those of the RP and LP groups, respectively. However, no significant difference was observed among the three groups. The mean stiffness value of the LS group (17.8 ± 2.6 N/mm) was lower than those of the RP (20.2 ± 10.5 N/mm) (*P* = 0.937) and LP (21.8 ± 3.8 N/mm) (*P* = 0.093) groups; however, no significant difference was noted among the three groups.

**Table 1:** Failure force and stiffness of three fixation methods for horizontal oblique metacarpal shaft fractures

Parameters (unit)	Value	Three fixation approaches			
		2 LS	RP	LP	*P*†
Failure force (N)	Mean	78.5	69.3	68.2	0.135
	SD	6.6	17.6	14.2	
	Max	90.4	96.2	91.1	
	Min	70.7	53.5	50.7	
Stiffness (N/mm)	Mean	17.8	20.2	21.8	0.513
	SD	2.6	10.5	3.8	
	Max	23.3	40.4	29.1	
	Min	15.1	9.9	17.4	

*LS* Lag screws; *RP* Regular plate; *LP* Locking plate; *SD* Standard deviation; *Max* Maximum; *Min* Minimum

^†^Kruskal–Wallis test

## Discussion

**Fig. 5:**
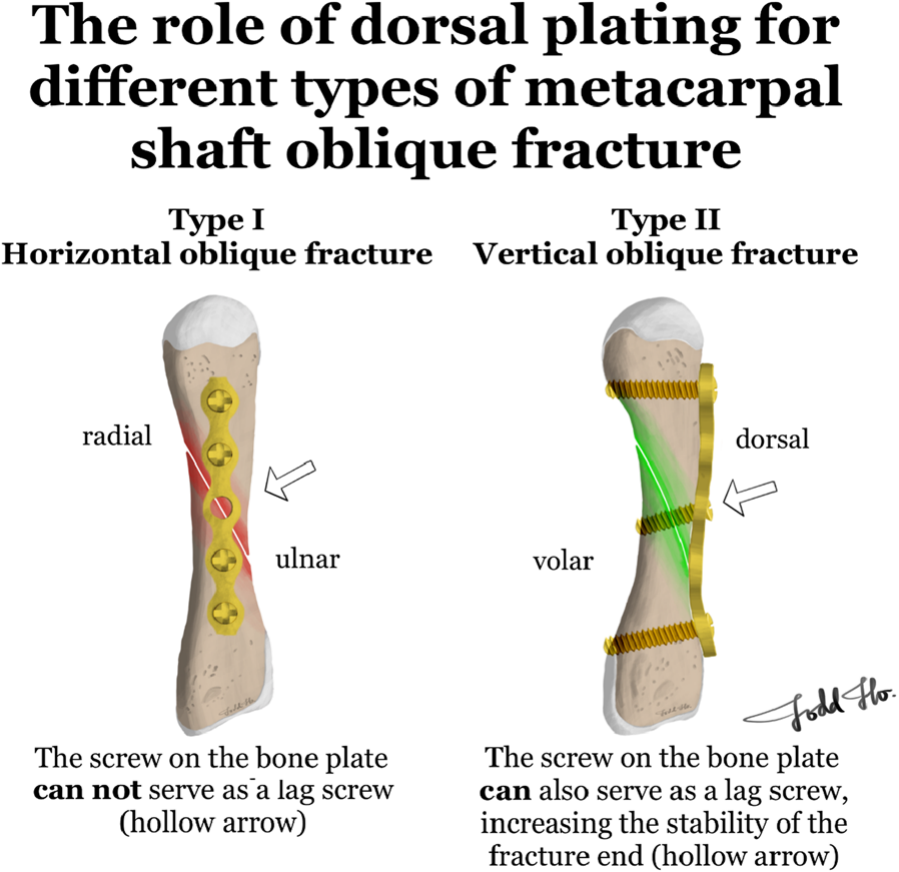
Bone screw on the dorsal plate cannot serve as a lag screw (white arrow) (left) and bone screw on the dorsal plate can serve as a lag screw (white arrow) (right)

In this study, we proposed a more detailed fracture classification based on whether the oblique fracture line mainly crosses the horizontal or vertical plane of the metacarpal shaft. Our results revealed that the fixation strength, measured by determining the effectiveness in sustaining force and stiffness, did not significantly differ among the LS, RP, and LP fixation. Treatment of these two types of oblique fractures differs as follows: (1) When dorsal bone plate fixation is used to fix type II fractures, the screw on the bone plate can serve as a lag screw, thus increasing the stability of the fracture end (Fig. 5 right). In type I fractures, the screw on the bone plate cannot serve as a lag screw. Therefore, the effectiveness of using bone plates to fix type I fractures is poorer (Fig. 5 left). (2) During hand prehension, the intrinsic muscles of the hand generate a bending force toward the metacarpal bone. Therefore, for type I fractures, the vector of the bending force can result in severe fracture site displacement. By contrast, for type II fractures, the vector of the bending force exerts a weaker effect in terms of causing fracture site displacement.

Metacarpal fractures are often caused by a direct blow during violence, axial loading due to falls on an outstretched hand, and torsion force from forceful traction [18]. Different injury mechanisms result in different fracture types. Fractures are typically categorized as transverse, oblique, spiral, or comminuted [18]. Vertical force and direct impact cause transverse or comminuted fractures, whereas torsion force results in oblique or spiral fractures. Among the different types of fracture, oblique and spiral are the most common, accounting for approximately 75% of all fractures [18]. The bone contact surface of the fracture site is small in transverse fractures. Thus, such fractures often result in nonunion due to the displacement or overlapping of the fractured bone end [4, 8]. This type of fracture usually requires surgical intervention. According to the literature and our previous mechanical study, favorable outcomes (fixation strength) can be achieved using both bone plate and intramedullary screw fixation [19–21]. The use of lag screws in transverse bone fractures is not favorable because they do not result in satisfactory fixation strength [22]. When bone plate fixation is performed to treat oblique fractures, bone plates of longer lengths are required to cover the longer fracture zone and ensure that screws can be fixed on the uninjured bone end. This is associated with extensive soft tissue dissection and postsurgical complications, including tendon adhesion, scar contracture, and joint stiffness [23]. By contrast, the use of lag screws to treat oblique metacarpal shaft fractures has resulted in favorable outcomes and does not require extensive soft tissue dissection around the fracture site [19, 20]. Treatment is generally effective when lag screw fixation is performed. However, biomechanical studies on lag screw fixation, especially those comparing the effectiveness of lag screw fixation with that of bone plate fixation in treating oblique metacarpal shaft fractures, are rare. Therefore, we proposed a more detailed fracture classification based on the fracture pattern and compared the fracture fixation effectiveness of lag screws and bone plates by using this classification.

In the studies on metacarpal fracture fixation, Chiu et al. [19, 24] used the same artificial metacarpal bone and cantilever bending biomechanical test. They used the headless compression screw, plate, and regular plate to fix the fracture in the middle of the metacarpal diaphysis. For the three fixation methods, the maximum fracture force values were 285.6, 227.8, and 228.2 N, respectively, and the stiffness values were 65.2, 61.7, and 54.9 N/mm, respectively. In addition, Chiu et al. [25] indicated that the use of the lag screw, regular bone plate, and locking plate to fix metacarpal vertical oblique shaft fractures resulted in maximum fracture force values of 153.6, 211.6, and 227.5 N, respectively, and stiffness values of 57.0, 64.7, and 65.4 N/mm, respectively. The findings of the previous study [25] and those of the current study indicate that the fixation strength of lag screws is not inferior to that of a metallic plate in either horizontal or vertical oblique fractures.

**Fig. 6:**
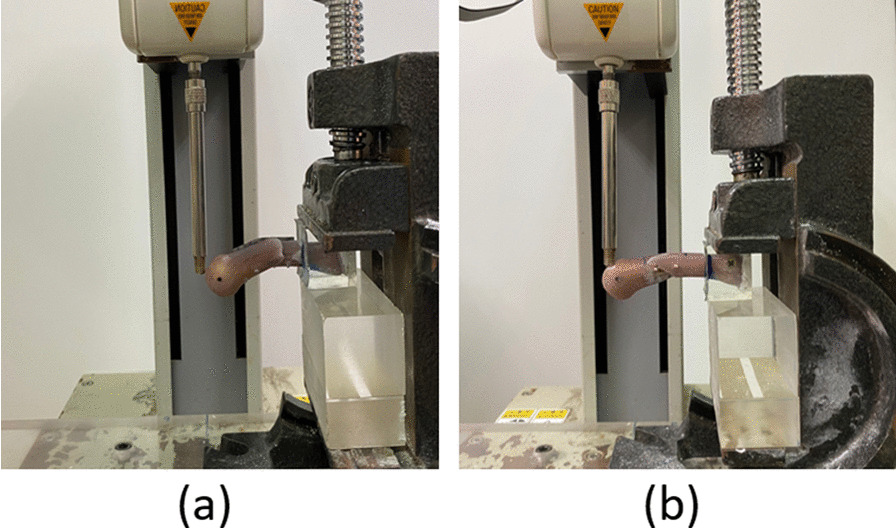
**a** The locking plate specimen was placed on the material testing system, and the fixture on which force was applied slipped off the force-bearing point of the artificial metacarpal bone. **b** Although the locking plate was permanently deformed, a fracture did not occur in the specimen

We used stiffness as an indicator to determine the fixation strength. However, we did not adopt the maximum fracture force as an indicator because when the material testing system was used to apply force on the distal region of the dorsal side of the artificial metacarpal bone in the RP and LP fixation, the maximum force was not observed at the actual fracture site in the entire specimen, as determined by plotting the force–displacement curve. Instead, the maximum force was observed at the point where the fixture on which force was applied slipped off the artificial metacarpal bone (Fig. 6a). At this time, the bone plate was already inserted into the plastic deformation region. Therefore, for the RP and LP fixation, the force value corresponding to the yield point was considered appropriate as the evaluation index, and we used it to represent the failure force. In the LS fixation, the samples exhibited an actual fracture and loosening, as determined from the force–displacement curve (Fig. 6b).

The surgical methods most commonly used in clinical practice to treat oblique metacarpal shaft fractures are: (1) lag screw fixation, (2) bone plate fixation, and (3) K-wire fixation [6]. Among these methods, bone plate fixation is generally considered to result in the strongest fixation effect; however, it requires longer surgical incisions and more extensive soft tissue dissection and is more expensive [9, 26]. For K-wire fixation, the fixation strength is insufficient to withstand the torsion load at the fracture site; this can lead to the rotational malunion of the fracture and eventually a scissoring deformity [2]. Therefore, most hand surgeons do not consider K-wire fixation to be suitable for the treatment of oblique metacarpal fractures. Lag screw fixation is a less invasive surgical procedure and typically results in favorable outcomes [27]. However, the uncertain mechanical stability of fixing by using only lag screws and the precise surgical technique required to place lag screws are major concerns for surgeons [27]. However, because the bone contact area of oblique metacarpal shaft fractures is relatively large, the bone healing potential for such fractures is higher than that for transverse fractures. Lag screw fixation can be more favorable for a larger fracture zone in oblique fractures. In addition to not causing complications normally associated with bone plate fixation, lag screw fixation involves shorter surgical incisions, less soft tissue dissection, lower costs, and shorter operating times [19, 20, 28].

This study has several limitations. First, we used artificial bone instead of human bone because of difficulty procuring fresh human metacarpal bones. Furthermore, even if such fresh bones were obtained, ensuring that all specimens possessed similar material properties would be impossible. Therefore, similar to most studies [1, 7, 20, 29], we used artificial bone instead of human metacarpal bone. Second, we performed the cantilever bending test to evaluate the effectiveness of different fixation methods for horizontal oblique metacarpal shaft fractures; this method was similarly used in other studies [19, 20, 30]. However, the movement of this loading model differs from that of real hands. Therefore, additional comprehensive experiments must be conducted to gain a better understanding of this topic. Third, compared with a true oblique fracture, a spiral-type oblique fracture is more commonly encountered in a real clinical scenario. However, in a biomechanical study, a true oblique fracture can be more easily reproduced and standardized in artificial metacarpal bone, and we can obtain study results with high reliability. Although we examined true oblique fractures in this study, we will focus on spiral-type oblique fractures in our future study.

## Conclusion

The fixation strength of two lag screw fixation did not significantly differ from that of regular bone plate and locking bone plate fixation, as indicated by the measurement of the ability to sustain force and stiffness. Because of the disadvantages of bone plate fixation, clinicians should consider two lag screw fixation as the primary surgical treatment for treating horizontal oblique metacarpal shaft fractures.

## Data Availability

All data generated or analyzed during this study are included in this published article.
